# The association between preoperative lacunar infarcts and postoperative delirium in elderly patients undergoing major abdominal surgery: a prospective cohort study

**DOI:** 10.1007/s40520-024-02909-1

**Published:** 2025-01-29

**Authors:** Danni Li, Pan Gu, Yuhao Wang, Yuchen Yao, Dan Fan

**Affiliations:** 1 Department of Anesthesiology, Sichuan Provincial People’s Hospital, School of Medicine, University of Electronic Science and Technology of China, Chengdu, 610000 Sichuan China; 2 Department of Anesthesiology, The Second People’s Hospital of Meishan City, Meishan, 620000 Sichuan China; 3 State Key Laboratory of Oral Diseases and National Center for Stomatology and National Clinical Research Center for Oral Diseases and Department of Oral and Maxillofacial Surgery, West China Hospital of Stomatology, Sichuan University, Chengdu, 610041 Sichuan China

**Keywords:** Lacunar infarcts, Postoperative delirium, Regional cerebral oxygen saturation, Elderly patients, Major abdominal surgery

## Abstract

**Objective:**

The primary goal was to investigate whether the presence of preoperative lacunar infarcts (LACI) was associated with postoperative delirium (POD) in elderly patients undergoing elective major abdominal surgery.

**Design:**

A prospective cohort study.

**Setting and participants:**

Patients aged ≥ 65 years from a tertiary level A hospital in China.

**Methods:**

The POD was assessed once daily within the first postoperative 3 days using the Confusion Assessment Method. Neurocognitive tests using the Mini-mental State Examination (MMSE) and the Beijing version of the Montreal Cognitive Assessment scales were carried out within 3 days before surgery and 4–7 days after surgery. Regional cerebral oxygen saturation (rScO_2_) was recorded in the operating room. Logistic regression analysis was used to evaluate the impact of preoperative LACI on POD and to explore the risk factors for POD.

**Results:**

A total of 369 participants were analyzed, 161 in the preoperative LACI-positive group (P group), and 208 in the preoperative LACI-negative group (N group), respectively. The incidence of POD was 32.7% in our study. The incidence of POD was significantly higher in the P group than in the N group (39.1 vs 27.9%, risk ratio, 1.66; 95% CI 1.07–2.58; *P* = 0.022). Furthermore, the P group exhibited lower mean rScO_2_ values during the procedure (*P* < 0.001). In exploratory analysis, the advanced age (*P* = 0.005), sex (*P* = 0.038), and lower preoperative MMSE score (*P* = 0.019) were independent risk factors for POD in patients undergoing major abdominal surgery.

**Conclusions and implications:**

Preoperative LACI was common, and constituted a risk factor for POD in older patients undergoing abdominal surgery. Despite the frequent subclinical nature, the preoperative LACI led to lower mean rScO_2_ during the procedure. These findings could help early identification of high-risk POD patients.

**Supplementary Information:**

The online version contains supplementary material available at 10.1007/s40520-024-02909-1.

## Introduction

In only 30 years’ time, the number of elderly individuals has escalated by 90% [1] . It is predicted that a doubling in the proportion of older adults will be achieved in 2050 [2] , predominantly in China, with an estimated 26.9% of the population expected to be aged over 65 [3]. The advances in anesthetic and surgical techniques are enabling more elderly individuals to undergo surgery. Consequently, a corresponding rise in postoperative delirium (POD), one of the most common and severe perioperative neurocognitive disorders among elderly surgical patients, is anticipated.

Major abdominal surgery is frequently performed in elderly patients, with POD occurring in up to 43.8% of cases [4] . As widely recognized, POD is linked to prolonged hospitalization [5] , higher 30 days readmission, dementia [6], and morbidity and mortality [7], which exacerbates the socioeconomic and healthcare burden caused by aging. Therefore, early recognition of patients at higher risk for POD has been pivotal in preventing and managing this condition, as well as optimizing postoperative outcomes.

Lacunar infarcts (LACI) refers to the presence of radiographically detected small cerebral ischemic lesions (< 15 mm diameter) caused by the occlusion of penetrating arteries in the deep brain structure but lack of clinical signs and symptoms [8] . Wide use of brain imaging has facilitated detection of LACI, which occurs in 20–50% of older adults [9, 10] . Since LACI presents without known clinical manifestations, it is increasingly acknowledged as a “silent killer” of cognitive functions in the elderly, contributing to vascular dementia [11], long-term cognitive decline [12, 13], and an increased risk of large vessel stroke [13–15]. However, it is still unclear whether postoperative neurological injuries occurred for patients with preoperative LACI undergoing abdominal surgery.

Regional cerebral oxygen saturation (rScO_2_) is a non-invasive, real-time, and continuous technique that employs near-infrared spectroscopy technology to assess the balance between cerebral oxygen consumption and delivery [16]. A systematic review found that perioperative rScO_2_ values below 50–60% or decreases exceeding 20% from the baseline were associated with postoperative neurological complications [17]. In acute ischemic stroke patients, preoperative cerebral oximetry often served as a predictor of neurological outcomes [18, 19]. However, the relationship between a chronic, pre-existing LACI and intraoperative rScO_2_ is not well understood and the predictive role of rScO_2_ for POD in patients with preoperative LACI is unclear.

Therefore, we sought to examine the primary hypothesis that preoperative LACI would correlate with POD within 3 days post-abdominal surgery among elderly patients and explore the impact on POD imposed by preoperative LACI. Furthermore, our secondary aim was to inform the relationship between preoperative LACI and intraoperative rScO_2_.

## Methods

### Study design

This single-center, prospective, observational cohort study was registered before enrollment at chictr.org.cn (ChiCTR2100054497, date of registration: December 18, 2021, principal investigator: Dan Fan) and approved by Ethics Committee of Sichuan Academy of Medical Sciences and Sichuan Provincial People’s Hospital (Number 2022304). The study was conducted in the Department of Anesthesiology, Sichuan Provincial People’s Hospital University of Electronic Science and Technology of China. Written informed consent was obtained from all subjects before entering the trial. The study adheres to the Strengthening the Reporting of Observational Studies in Epidemiology (STROBE) guidelines.

### Participants

Patients aged ≥ 65 years scheduled to undergo a 2-h-or-longer abdominal surgery between January 1, 2022 and November 7, 2023 who were expected to remain hospitalized for 4 or more nights were eligible. The exclusion criteria were: (1) American Society of Anesthesiologists Physical Status (ASA) IV or higher; (2) History of mental disease and neurologic disorders; (3) Drug or alcoholism dependency; (4) Preoperative cognitive dysfunction: identified using education-specific cutoff points of total Mini-Mental State Examination (MMSE) score: ≤ 17 for illiteracy, ≤ 20 for less than primary education, and ≤ 23 for less than a post-secondary education; [20] (5) Acute stroke within 3 months (6) Refusal to participate.

The primary exposure in this study was preoperative LACI. The identification of LACI was assessed by magnetic resonance imaging (MAGNETOM vida, 3.0 Telsa, Siemens Healthineers, Germany) before surgery. The assessment was conducted by a panel of experts not involved in the study. These patients had confirmed preoperative LACI and were subsequently allocated to the preoperative LACI-positive group (P group), or the preoperative LACI-negative group (N group).

### Intraoperative management

#### Preoperative preparation

All patients were grouped preoperatively by cerebral magnetic resonance imaging. 1–3 days before surgery, the preoperative cognitive status was assessed with the MMSE and the Beijing version of the Montreal Cognitive Assessment Scale (MoCA-BJ) [21].

#### Anesthesia and perioperative care

Typical anesthesia induction was performed. During the operation, blood pressure was maintained within the range of ± 20% of the baseline. There were no limitations for vasoactive medications except for the avoidance of hormones, long-acting sedatives, and dexmedetomidine. Blood gas analysis was performed for intra-period: (1) T_0_: at room air; (2) T_1_: at 1 h after surgery; (3) T_2_: at 2 h after surgery; (4) T_3_: at the end of surgery. The pain assessment using the Numerical Rating Scale (NRS) [22] was conducted during the first postoperative 3 days. If the pain score ≥ 6, the patient was withdrawn from the trial. The detailed anesthesia plan was described in the protocol.

#### Cerebral oxygen saturation monitoring

Cerebral oxygen saturation was monitored using the Enginmed EGOS-600 cerebral oximeter (EGOS-600B; Enginmed Bio-Medical Electronics, Suzhou, China). Cerebral oxygen saturation was monitored after entering the operating room and continued until the end of the surgery. The cerebral oximeter generated data every 2 s, which was extracted from the monitor at the end of surgery. Variables including baseline data and patient characteristics with more than 20% missing were excluded from the analysis. The left and right rScO_2_ values were averaged, and these mean values were used for calculating rScO_2_-related indicators.

### Outcomes and definitions

#### Assessment of delirium

POD was assessed once daily during 18:00–20:00, using the Chinese version of the Confusion Assessment Method (CAM) in non-intubated patients and the Confusion Assessment Method for the Intensive Care Unit in intubated patients [23]. Neurocognitive tests using the MMSE and MoCA-BJ scales were carried out within 3 days before surgery and 4–7 days after surgery. Patients were evaluated by two experienced research personnel independently. POD was identified if patients were positive with CAM testing at least once within the first 3 days after surgery. Delayed neurocognitive recovery (DNR) was diagnosed using the MoCA assessments [24, 25]. The MoCA scales were performed on postoperative day 4 to 7. A Z score was obtained to determine the incidence of DNR [24, 25]. The mean practice effect, defined as the mean change in MoCA score of control subjects (ΔX_control_), was determined by comparing the test score of control subjects before and after non-surgical interventions. We selected 20 patients with gastrointestinal malignancies who received neoadjuvant chemotherapy immunotherapy or radiotherapy as the control group. Their inclusion criteria were similar to surgical patients. The difference between pre-and post-MoCA score (ΔX) subtracts the average learning effect in the control group (ΔX − ΔX_control_). The result was then divided by the standard deviation of the control group (SD_ΔXcontrol_). The DNR was diagnosed when the Z score of an individual was 1.96 or greater. The equation was Z = (ΔX − ΔX_control_)/SD_ΔXcontrol_.

#### Cerebral oxygen saturation

Baseline measurements were obtained from patients in the supine position while breathing room air. We selected a 1 min epoch before the induction of anesthesia as baseline rScO_2_ value. Additionally, we examined the absolute rScO_2_ value at different phases of general anesthesia, including both induction and maintenance. We also calculated the relative desaturation, as its potential contribution to delirium [26]. Based on the literature, we selected rScO_2_ levels of ≤ 80%, 85%, 90% of baseline as the relative thresholds when calculating the area under the threshold (AUT) and AUT percentages (defined as the proportion of the area below the threshold to the total area). Given that studies investigating the relationship between intraoperative cerebral desaturation and adverse outcomes often set the threshold at a 20% drop from baseline rScO_2_ [17], a cerebral desaturation event in this study was defined as a reduction in rScO_2_ values by 10%, 20% or more from baseline, lasting for 1 min or longer. Subsequently, dynamic changes of rScO_2_ were recorded at different stages: (1) T_0_: at room air; (2) T_1_: at the start of induction; (3) T_2_: at the end of induction; (4) T_3_: at the start of surgery; (5) T_4_: at 1 h after surgery; (6) T_5_: at 2 h after surgery; (7) T_6_: at the end of surgery.

#### Other outcome assessments

Intraoperative blood gas analysis, anesthetics, and vasoactive drugs were recorded. Blood gas analysis was performed for measurement of arterial partial pressure of oxygen, arterial partial pressure of carbon dioxide, hemoglobin (Hgb), hematocrit, and lactate levels. We also observed the postoperative pain score via NRS and adverse outcomes.

### Statistical analysis

The sample size was calculated using PASS 15.0 (NCSS Statistical Software, East Kaysville, USA). The primary outcome of this trial was the incidence of POD. The sample size requirement was estimated based on the following assumptions: (1) about 50% incidence of preoperative LACI [9, 10, 27]; (2) 30% and 45% POD in patients with versus without preoperative LACI based on the previous literature [4, 30]; (3) Power of 80% and a two-sided significance level of 0.05; (4) 5% dropout rate. A total sample size of 336 patients was required in this study, 168 in each group, respectively.

The SPSS 26.0 software (IBM, Armork, USA) was used for data analysis. Normality was tested using the Kolmogorov–Smirnov test. Continuous variables with normal distribution were presented as mean ± standard deviation, while those with a non-normal distribution were appropriately reported as median (25th, 75th percentiles) or median [minimum, maximum]. Categorical variables were presented as frequency (column %). The continuous data with a normal distribution were analyzed using the Student’s *t* test, while those not following normality were compared using the Mann Whitney *U* test. Categorical data were compared with a *χ*^2^ test or Yates continuity-corrected *χ*^2^ test. A 2-tailed *P* < 0.05 was considered statistically significant.

The logistic regression model was constructed using backward stepwise selection to examine the association of patient characteristics and perioperative variables with the occurrence of POD. Given the relevant literature, We entered the potential determinants, including the presence of preoperative lacunar infarcts, sex [28, 29], age [30, 31], preoperative MMSE [30], education level [32, 33], hypoalbuminemia [30], anemia [2, 30, 31], ASA physical status [30, 31, 34], duration of surgery [31, 34], cerebral desaturation [35–37], and baseline rScO_2_ [35–37] into the model. We applied stepwise regression using likelihood-ratio tests and backward elimination to construct the model. The cut-off for variable removal was set at a significance level of 0.1.

## Results

### Patients screening and baseline characteristics of patients

During the study period, the final analysis included 369 patients, with 161 patients in the P group and 208 patients in the N group, respectively (Supplementary Fig. 1). The baseline characteristics of the included patients are shown in Table 1. The incidence of preoperative LACI was 43.6% (161/369) in this cohort. Patients with preoperative LACI were older (*P* = 0.010) and had a higher ASA classification (*P* = 0.015, Table 1). Additionally, they required more ephedrine (*P* = 0.014) and had lower postoperative MoCA score (*P* = 0.018, Table 1). No significant differences were observed in other perioperative variables.

**Table 1:** Basic demographic characteristics and anesthesia/surgical data

Variables	Overall	N group (n = 208)	P group (n = 161)	*P* value
Preoperative variables				
Age, y	71 (66, 75)	70 (66, 74)	72 (68, 76)	0.010
Age classification, n (%)				
65–75	261 (70.7%)	157 (75.5%)	104 (64.6%)	0.074
75–85	102 (27.6%)	48 (23.1%)	54 (33.5%)	
≥ 85	6 (1.6%)	3 (1.4%)	3 (1.9%)	
Male	257 (69.6%)	108 (67.1%)	149 (71.6%)	0.345
BMI (kg/m^2^)	23.1 ± 2.9	23.1 ± 2.9	23.0 ± 2.9	0.584
Education level				
Primary school or below	176 (47.7%)	94 (45.2%)	82 (50.9%)	0.355
Middle school	120 (32.5%)	74 (35.6%)	46 (28.6%)	
High school or above	73 (19.8%)	40 (19.2%)	33 (20.5%)	
Comorbidity, n (%)				
Hypertension	200 (54.2%)	111 (53.4%)	89 (55.3%)	0.714
Diabetes	68 (18.4%)	38 (18.3%)	30 (18.6%)	0.929
Coronary artery disease	54 (14.6%)	28 (13.5%)	26 (16.1%)	0.469
COPD	66 (17.9%)	42 (20.2%)	24 (14.9%)	0.189
Obesity^a^	4 (1.1%)	3 (1.4%)	1 (0.6%)	0.804
Anemia^b^	116 (31.4%)	59 (28.4%)	57 (35.4%)	0.149
Hypoalbuminemia^c^	14 (3.8%)	6 (2.9%)	8 (5.0%)	0.299
Renal function damage^d^	12 (3.3%)	6 (2.9%)	6 (3.7%)	0.651
ASA ≥ III	255 (69.1%)	133 (63.9%)	122 (75.8%)	0.015
Cognitive impairment^e^	29 (7.9%)	15 (7.2%)	14 (8.7%)	0.599
Preoperative MMSE	28 (27, 29)	28 (27, 29)	28 (27, 29)	0.266
Preoperative MoCA	21 (19, 24)	22 (19, 24)	21 (19, 23)	0.082
Hemoglobin (g/dL)	122 (105, 135)	123 (108, 137)	119 (100, 134)	0.079
Albumin (g/L)	39.8 (37.0, 42.6)	40.2 (37.7, 42.7)	39.5 (35.7, 42.4)	0.084
Creatinine (μmol/L)	75.0 (64.0, 86.2)	74.6 (64.0, 85.0)	76.0 (62.8, 88.0)	0.648
Glucose (mmol/L)	5.0 (4.5, 5.8)	5.0 (4.5, 5.6)	5.1 (4.5, 6.0)	0.381
Intraoperative variables				
Laparoscopic approach, n (%)	248 (67.2%)	144 (69.2%)	104 (64.6%)	0.347
Sufentanil dose (ug)	30 (25, 35)	30 (26, 35)	30 (25, 35)	0.466
Vasoactive drug				
Ephedrine				
No. received, n (%)	272 (73.7%)	143 (68.8%)	129 (80.1%)	0.014
Dose (mg)	12[0, 36]	9[0, 36]	12[0, 36]	0.015
Phenylephrine				
No. received, n (%)	11 (3.0%)	6 (2.9%)	5 (3.1%)	1.000
Dose (mg)	0[0, 6.5]	0[0, 0.5]	0[0, 6.5]	0.894
Norepinephrine				
No. received, n (%)	50 (3.5%)	27 (13.0%)	23 (14.3%)	0.716
Dose (mg)	0[0, 4.8]	0[0, 4.8]	0[0, 4.1]	0.843
Atropine				
No. received, n (%)	333 (90.2%)	186 (89.4%)	147 (91.3%)	0.335
Dose (mg)	0.5 (0.5, 0.5)	0.5 (0.5, 0.5)	0.5 (0.5, 0.5)	0.089
Crystalloid infusion (ml)	1500 (1100, 1800)	1500 (1100, 1800)	1500 (1100, 1900)	0.601
Colloid infusion (ml)	500 (400, 750)	500 (400,700)	500 (400, 800)	0.615
Urine output (ml)	300 (200, 525)	300 (200, 500)	350 (200, 550)	0.485
Blood variables				
Lowest hemoglobin (g/dL)	96 (84, 107)	96 (84, 107)	96 (81, 107)	0.430
Lowest hematocrit	0.31 (0.27, 0.34)	0.31 (0.27, 0.34)	0.30 (0.26, 0.34)	0.229
Lowest PaO_2_ (mmHg)	203 (171, 230)	202 (176, 228)	205 (169, 235)	0.691
Lowest PaCO_2_ (mmHg)	42 (39, 46)	42 (39, 46)	42 (38, 46)	0.433
Highest lactate (mmol/L)	0.8 (0.7, 1.1)	0.8 (0.6, 1.1)	0.8 (0.7, 1.1)	0.386
Duration of surgery (h)	3.7 (2.7, 4.8)	3.8 (2.8, 5.0)	3.4 (2.6, 4.8)	0.174
Duration of anesthesia (h)	4.3 (3.3, 5.5)	4.4 (3.3, 5.6)	4.1 (3.2, 5.3)	0.150
Postoperative variables				
Postoperative MMSE	27 (26, 28)	27 (26, 28)	27 (26, 28)	0.381
Postoperative MoCA	20 (18, 22)	21 (19, 22)	20 (18, 22)	0.018
Maximum NRS score	3 (2, 4)	3 (2, 4)	3 (3, 4)	0.527
Postoperative hospital stay (d)	9 (7, 13)	9 (7, 12)	9 (8, 14)	0.121

Data are presented as mean ± standard deviation, median (25th, 75th percentiles), median [min, max], or frequency (column %)

*BMI* body mass index, *COPD* chronic obstructive pulmonary disease, *ASA* American Society of Anesthesiologists physical status, *MMSE* Mini-Mental State Examination, *MoCA* Montreal Cognitive Assessment, *NRS* numeric rating scale, *N* preoperativelacunar infarcts-negative group, *P* preoperativelacunar infarcts-positive group

^a^Obesity is defined as BMI ≥ 30 kg/m^2^

^b^Anemia is defined as hemoglobin ≤ 110 g/L

^c^Hypoalbuminemia is defined as albumin ≤ 30 g/L

^d^Renal function damage is defined as creatinine ≥ 133 μmol/L

^e^Cognitive impairment is defined as a MMSE score ≤ 24

### Preoperative lacuna infarcts and postoperative delirium

The incidence of POD was 32.7% (121/369). The incidence of POD was significantly higher in the P group than in the N group (39.1 vs 27.9%, risk ratio, 1.66; 95% CI 1.07–2.58;* P* = 0.022, Table 2). Compared with patients in the N group, the POD incidence in the P group was significantly higher on postoperative 3 days (all *P* < 0.05, Table 2). There were no significant differences in the incidence of DNR between the two groups (*P* = 0.598, Table 2).

**Table 2:** Neurocognitive outcomes

	Overall	N group (n = 208)	P group (n = 161)	Risk ratio (95% CI)	*P* value
POD	121 (32.7%)	58 (27.9%)	63 (39.1%)	1.66 (1.07–2.58)	0.022
Postoperative day 1	104 (28.1%)	44 (21.2%)	60 (37.3%)	2.21 (1.40–3.51)	0.001
Postoperative day 2	98 (26.5%)	46 (22.1%)	52 (32.3%)	1.68 (1.06–2.68)	0.028
Postoperative day 3	78 (21.1%)	36 (17.3%)	42 (26.1%)	1.69 (1.02–2.79)	0.041
DNR	71 (19.2%)	42 (20.2%)	29 (18.0%)	0.87 (0.51–1.47)	0.598

Data are presented as frequency (column %)

*POD* postoperative delirium, *DNR* delayed neurocognitive recovery, *CI* confidence interval, *N* preoperativelacunar infarcts-negative group, *P* preoperativelacunar infarcts-positive group

### Preoperative lacunar infarcts and regional cerebral oxygen saturation

**Fig. 1:**
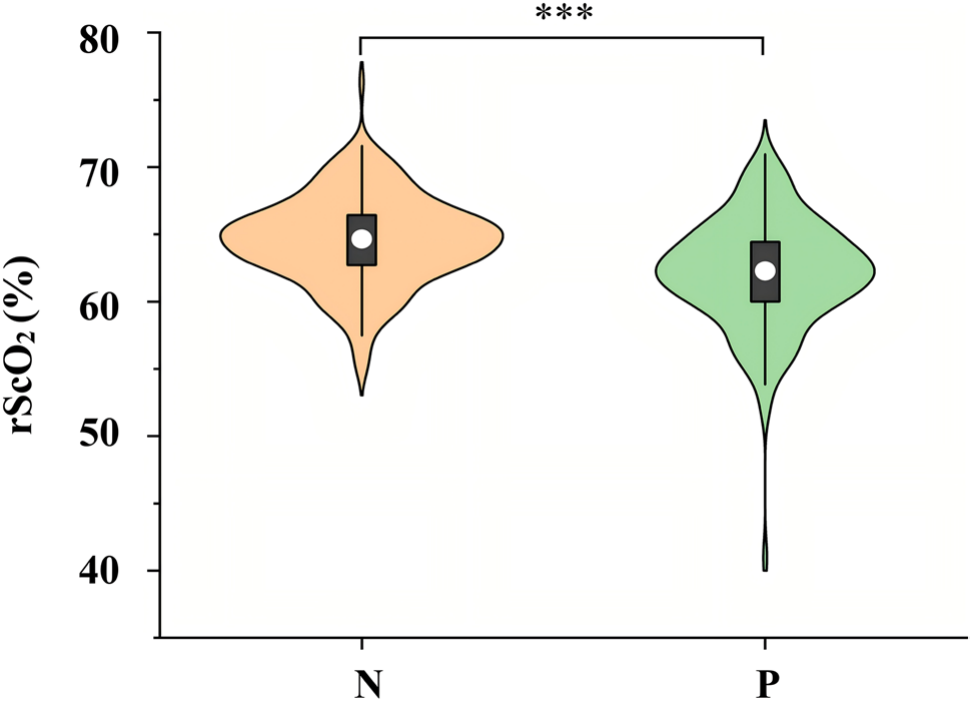
Violin plot of average rScO_2_ in N and P groups. rScO_2_, regional cerebral oxygen saturation; N, preoperative lacunar infarcts-negative group; P: preoperative lacunar infarcts-positive group. The white dot indicated the median, the black bar the interquartile range, and the whiskers the range, respectively. A kernel density estimation outlines the shape of the rScO_2_ distribution. The wider sections reflect where more data points are concentrated, indicating that higher probability for corresponding rScO_2_ values, whereas narrower sections suggest a lower probability of occurrence. The left and right rScO_2_ values were averaged and the average rScO_2_ was monitored after entering the operating room and continued until the end of the surgery. ***P < 0.001

The P group had lower mean rScO_2_ than the N group during the pre-and intraoperative period (all *P* < 0.001, Table 3). The detailed distribution of mean rScO_2_ for the N and P groups was presented in Fig. 1. Higher mean rScO_2_ at different times were observed in the N group at all time points during the surgery **(**Supplementary Fig. 2). A significant increase in rScO_2_ values was observed during induction of anesthesia. After induction of anesthesia, changes in rScO_2_ plateaued. Both groups experienced a similar desaturation. The AUTs of rScO_2_ ≤ 80%, 90% of baseline was significantly larger in the P group than in the N group (all *P* < 0.05, Table 3). The incidence of cerebral desaturation (dropped by 10% or more from baseline) was higher in the P group (*P* = 0.042, Table 3). The AUT percentages did not differ between two groups (Table 3).

**Table 3:** Regional cerebral oxygen saturation values

rScO_2_	N group (n = 208)	P group (n = 161)	*P* value
Baseline rScO_2_	62.2 (60.5, 64.3)	60.3 (57.8, 62.7)	< 0.001
Induction^a^	64.2 (62.1, 66.2)	62.2 (59.7, 64.4)	< 0.001
Maintenance^b^	64.8 ± 3.5	62.4 ± 4.1	< 0.001
Cereral desaturation 10%^c^	26 (16.1%)	19(9.1%)	0.041
Cereral desaturation 20%^d^	4 (1.9%)	8 (5.0%)	0.102
AUT of rScO_2_ (% × min)			
< 80% of baseline	0 [0, 60]	0 [0, 1456]	0.022
< 85% of baseline	0 [0, 478]	0 [0, 2042]	0.121
< 90% of baseline	0 [0, 1574]	0 [0, 2672]	0.004
AUT percent^e^ (%)			
< 80% of baseline	0 [0, 0.15]	0 [0, 0.82]	0.293
< 85% of baseline	0 [0, 0.53]	0 [0, 0.93]	0.494
< 90% of baseline	0 [0, 0.91]	0 [0, 1]	0.073

Data are presented as mean ± standard deviation, median (25th, 75th percentiles), median [min, max], or frequency (column %)

*AUT* area under the threshold

^a^Induction was defined as the period from the start of induction to the end of induction

^b^Maintenance was defined as the period from the end of induction to the end of surgery

^c^Cerebral desaturation was defined as a reduction in oximetry values by 10% or more from baseline, lasting for 1-min or longer

^d^Cerebral desaturation was defined as a reduction in oximetry values by 20% or more from baseline, lasting for 1-min or longer

^e^AUT percent was defined as the proportion of the area below the threshold to the total area

### Risk factors for POD

**Fig. 2:**
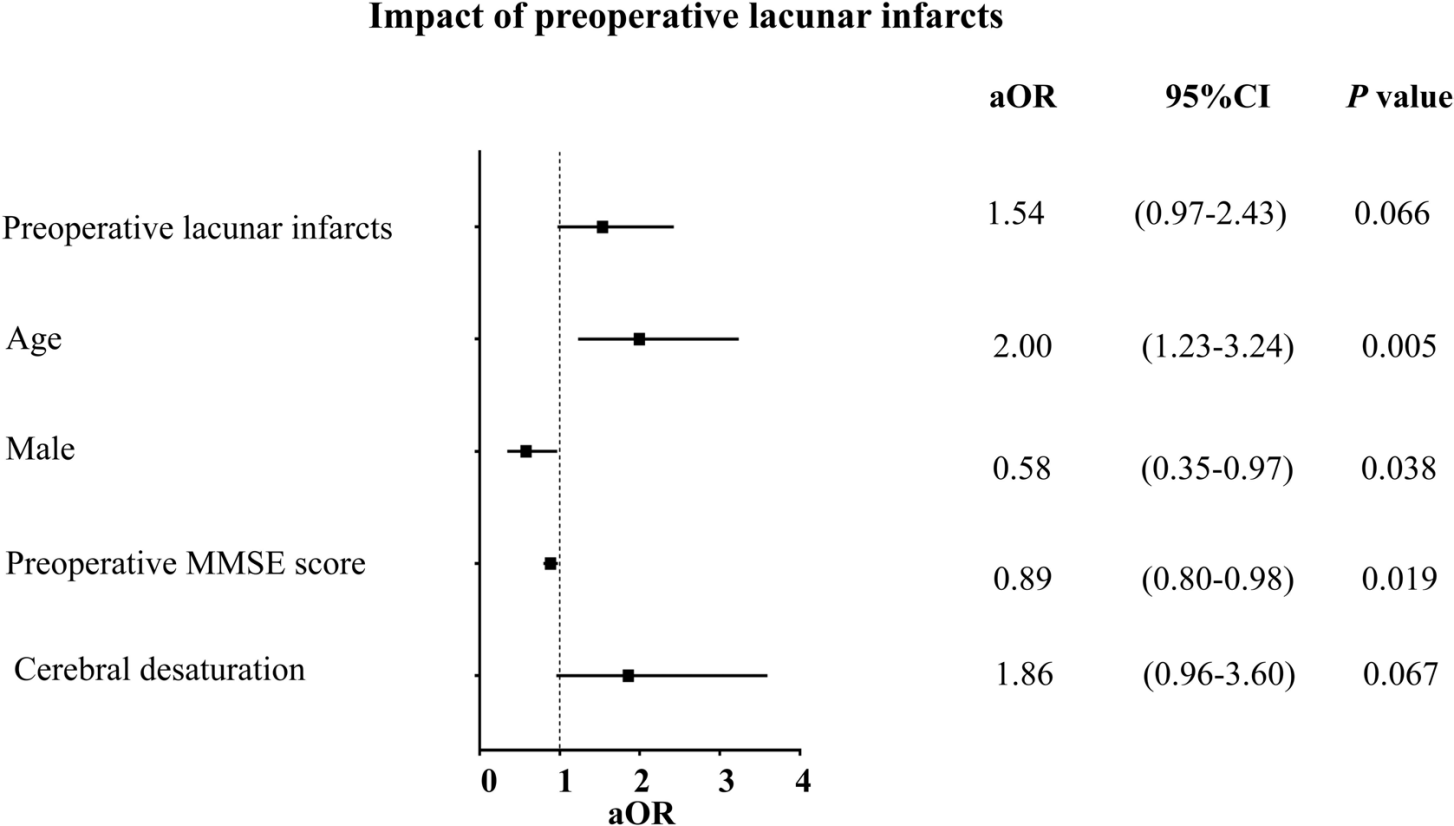
Impact of preoperative lacunar infarcts. MMSE, Mini-Mental State Examination; aOR, adjusted odds ratio; CI, confidence interval. Cerebral desaturation was defined as a reduction in oximetry values by 10% or more from baseline, lasting for 1-minute or longer

The logistic regression analysis of perioperative data was shown in Supplementary Table 3. Due to collinearity, the following variables were encluded in the multivariable analysis: anemia and lowest Hgb; preoperative MMSE and preoperative MoCA score; pre- and postoperative MMSE score, and pre-and postoperative MoCA score. The multivariable analysis reported that the advanced age [adjusted odds ratio (aOR), 2.00; 95% CI 1.23–3.24; *P* = 0.005], male [aOR, 0.58, 95% CI 0.35–0.97; *P* = 0.038] and lower preoperative MMSE score (aOR, 0.89, 95% CI 0.80–0.98; *P* = 0.019) remained independent risk factors for POD (Fig. 2, Supplementary Table 3).

### Other outcomes

No differences in intraoperative data and postoperative NRS score were observed between N and P groups in Supplementary Table 1.

## Discussion

The main finding of our study was that among the elderly patients who underwent major abdominal surgery, those with preoperative LACI experienced a higher incidence of POD (39.1 vs 27.9%). Lower rScO_2_ levels both before and during surgery in the P group compared with the N group were detected. Although anesthesia management for patients with fragile brain function remains challenging, the study highlighted older age, male, and lower preoperative MMSE scores were risk predictors for POD in these patients, providing a potential target for prevention.

In our cohort, the prevalence of preoperative LACI was 45.8%, indicating that the cognitive prognosis of this population deserves attention in the perioperative period. Previous studies have demonstrated that patients with preoperative covert brain infarcts were at a higher risk for postoperative cognitive dysfunction following cardiac surgery [38, 39]. However, these studies had failed to differentiate carefully between lacunar and cortical infarcts. Another trial suggested that preoperative lacunar infarcts were not associated with delirium [40]. Nevertheless, our trial has produced conflicting results. Such inconsistency may be explained by inadequate consideration of the extensive perioperative risk factors associated with POD (preoperative, intraoperative, and postoperative). No detailed description of the variety of anesthesia profiles was reported in the previous study. This could limit the accurate assessment of POD incidence in specific populations. Additionally, the extent and location of infarction lesions, reflective of cerebral small vessel disease severity, may deeply influence cognitive prognosis [12]. Further in-depth research will be conducted in the future. Symptomatic lacunes has been confirmed as an important predictor of long-term cognitive decline [41]. The **s**trength of our study is the focus on preoperative LACI, which primarily represents asymptomatic patients with a potential risk of exacerbating cognitive decline. This asymptomatic characteristic, leading to challenges in tracking the course of cognitive change, impacts the timely identification and effective intervention of neurocognitive disorders [42]. Prevention is better than cure, for currently there is no specific treatment available for delirium. Determining the association between preoperative LACI and POD supports the early identification of brain vulnerabilities through routinely collected data.

The overall incidence of POD in this study was 32.7%, which is higher than a meta-analysis and system review of adults following major abdominal surgery [30]. This discrepancy may stem from differences in the surgery type and the characteristics of the patient cohort. The major abdominal surgery itself was a known risk factor for POD [43–46] Moreover, advanced age (over 65 years) was a significant risk factor for POD [30–32, 47]. In our study, the median age of the patients was 71 years. Furthermore, as demonstrated in our research, lacunes led to cognitive impairment [41]. Those factors may partly explain the relatively high incidence of POD.

No significant differences in baseline cognition using MMSE and MoCA assessments were observed between the P and N groups, reflecting the covert clinical features of LACI. The LACI was featured as cerebral small vessel disease compared to other large vessel diseases such as cortical brain infarcts, thus the relatively mild nature of LACI did not act as cognitive impairment. Building upon subjective scale assessments, we combined sensitive and objective rScO_2_ measurements to enhance the evaluation of brain function. Our study provided the most detailed dataset at 2 s intervals to date. The patients in the P group exhibited lower rScO_2_ values than that in the N group. Patients with pre-existing brain infarcts tend to have a worse basic regional brain infusion [48]. Lower rScO_2_ variables in the surgery were further observed in patients with preoperative LACI, indicating that the cerebral function is highly vulnerable to hypoxemia due to a preoperative hypoperfusion state. Cerebral autoregulation impairment in cerebral ischemic infarctions has been validated in several studies [49, 50]. In LACI cohort, a certain cerebrovascular reserve capacity was impaired preoperatively. Moreover, the capacity of brain is further reduced due to weaker arterial elasticity and other comorbidities in the elderly. When surgical stimulation causes circulatory fluctuations that exceed the compensatory capability of brain, cerebral blood flow decreases proportionally with perfusion pressure, resulting in ischemia and hypoxia and subsequently a decrease in rScO_2_ [51]. We speculated that the mechanism underlying LACI contributing to POD may involved with pre- and intraoperative impaired cerebral perfusion. Whether low intraoperative rScO_2_ mediated such association needs to be studied in the future.

Furthermore, we studied the impact of preoperative LACI on POD. A retrospective prediction model study of non-cardiac surgery summarized that the most influential predictors for POD, ranked by significance, were previous delirium, duration of anesthesia, and ASA class [34]. Anemia is common in major abdominal surgical patients [43, 44], and its impact on POD remained controversial [30], due to different definitions used. [2, 30, 31, 45] In China, preoperative anemia in adults was defined as Hgb < 110 g/L in females and Hgb < 120 g/L in males [52]. ASA classification, duration of surgery, and anemia were additional perioperative variables, which entered into the model beyond factors in backward logistic regression analysis. Following this, anemia, ASA classification, and duration of surgery were progressively eliminated to construct the optimal model. The ORs were both > 1 before and after revising the model, suggesting that preoperative LACI continued to affect adversely on POD.

In the exploratory analysis of risk factors for POD in this cohort, we found that these patients**,** who were of advanced age, male, and had lower preoperative MMSE score, faced an increasing risk of POD. These findings allow clinicians to identify high-risk POD patients easily, and allocate health resources efficiently. However, the predictive role of rScO_2_ for POD in these patients remains uncertain. There seems to be with need to further include more rScO_2_ variables through larger-scale, multi-center studies.

There were some limitations in our study. First, the trial was a single-center study, further multi-institutional research was needed. Second, the CAM assessment only once a day may have resulted in underdiagnosis. Third, we were unable to obtain pre- and postoperative MRI examinations to evaluate structural changes in the brain that result from anesthesia and surgery. Fourth, the lack of rScO_2_ data post-surgery limits our ability to explore the brain function changes during follow-up.

## Conclusions and implications

In summary, preoperative LACI increased the risk of POD in the initial 3 days after major abdominal surgery in the elderly. Although preoperative LACI was prevalent and often presented subtly, it had been confirmed to be associated with reduced rScO_2_ levels. Our study underlined the importance of involving preoperative LACI as a potential determinant in postoperative cognitive function assessment.

## Supplementary Information

Below is the link to the electronic supplementary material.

Supplementary Figure 1. Flow chart. ASA, American Society of Anesthesiologists Physical Status; rScO_2_, regional cerebral oxygen saturation; LACI, lacunar infarcts (JPG 86 KB)

Supplementary Figure 2. The changes of average rScO2 at different times. Data are presented as mean±standard deviation. rScO_2_, regional cerebral oxygen saturation. N, preoperative lacunar infarcts-negative group; P preoperative lacunar infarcts-positive group T_0_, at room air; T_1_, at the start of induction; T_2_, at the end of induction; T_3_, the start of surgery; T_4_, at 1 h after surgery; T_5_, at 2 h after surgery; T_6_, at the end of surgery. ***P < 0.001 (JPG 81 KB)

Supplementary file3 (PDF 271 KB)

## Data Availability

No datasets were generated or analysed during the current study.
