# Supplementary figures and images for: The association between preoperative lacunar infarcts and postoperative delirium in elderly patients undergoing major abdominal surgery: a prospective cohort study
Source: Aging Clin Exp Res. 2025 Jan 29;37(1):35. doi: 10.1007/s40520-024-02909-1 (PMC11779751; doi:10.1007/s40520-024-02909-1)

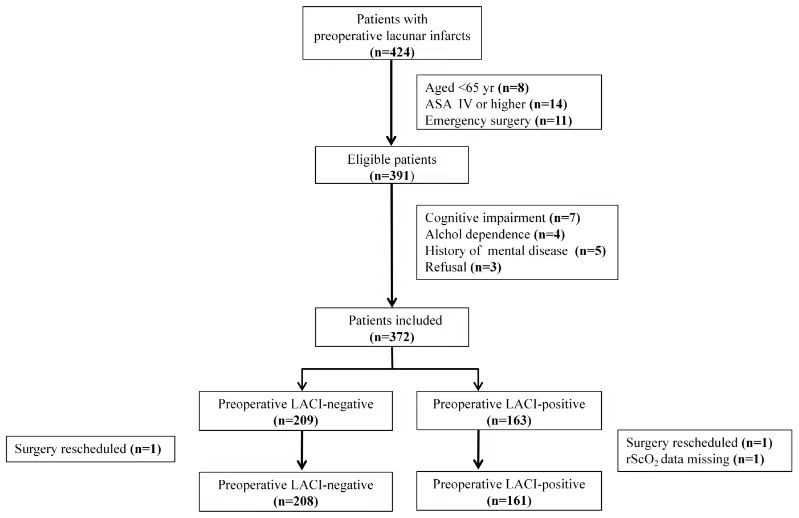

Supplement: Supplementary file 1 — Supplementary Figure 1. Flow chart. ASA, American Society of Anesthesiologists Physical Status; rScO2, regional cerebral oxygen saturation; LACI, lacunar infarcts (JPG 86 KB) [file 40520_2024_2909_MOESM1_ESM.jpg]

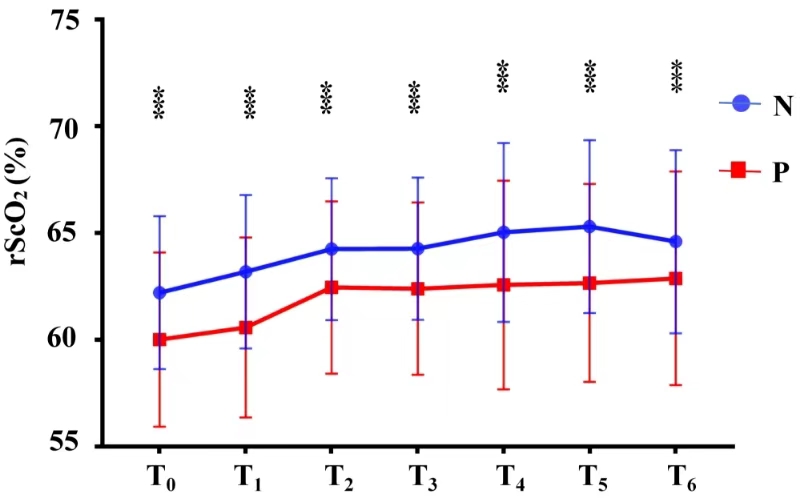

Supplement: Supplementary file 2 — Supplementary Figure 2. The changes of average rScO2 at different times. Data are presented as mean±standard deviation. rScO2, regional cerebral oxygen saturation. N, preoperative lacunar infarcts-negative group; P preoperative lacunar infarcts-positive group T0, at room air; T1, at the start of induction; T2, at the end of induction; T3, the start of surgery; T4, at 1 h after surgery; T5, at 2 h after surgery; T6, at the end of surgery. ***P < 0.001 (JPG 81 KB) [file 40520_2024_2909_MOESM2_ESM.jpg]
