# Supplementary material for: The association between preoperative lacunar infarcts and postoperative delirium in elderly patients undergoing major abdominal surgery: a prospective cohort study
Source: Aging Clin Exp Res. 2025 Jan 29;37(1):35. doi: 10.1007/s40520-024-02909-1 (PMC11779751; doi:10.1007/s40520-024-02909-1)
Supplement: Supplementary file 3 — Supplementary file3 (PDF 271 KB) [file 40520_2024_2909_MOESM3_ESM.pdf]

Supplementary Tables

Supplementary Table 1. Perioperative characteristics between N and P groups.

Supplementary Table 2. Dynamic changes of intraoperative regional cerebral oxygen saturation between N and P groups.

Supplementary Table 3. Backward logistics regression analysis of Model 1

Supplementary Table 4. Logistics analysis of Model 2

Supplementary Table 5. Logistics analysis of Model 3

Supplementary Table 6. Logistics analysis of Model 4

Supplementary Table 7. Adverse clinical outcomes during hospitalization

Supplementary Table 1. Perioperative characteristics between N and P groups.

Variables	Overall (n = 369)	N group (n = 208)	P group (n = 161)	*P* Value
Type of surgery				
Gastrointestinal Surgery	336(91.1%)	192(92.3%)	144(89.4%)	0.524
Hepatobiliary Surgery	20(5.4%)	10(4.8%)	10(6.2%)	
Urological surgery	12(3.3%)	5(2.4%)	7(4.3%)	
Obstetric surgery	1(0.3%)	1(0.5%)	0(0)	
Blood gas analysis				
T_0				
pH	7.41(7.37, 7.44)	7.40(7.36, 7.44)	7.41(7.37, 7.44)	0.176
Hgb	109(93, 121)	109(96, 121)	109(93, 121)	0.707
HCT	0.35(0.30, 0.39)	0.35±0.06	0.34±0.06	0.268
Lac	0.7(0.5, 0.9)	0.7(0.5, 0.9)	0.7(0.5, 0.9)	0.585
PaO_2	79(72, 86)	79(72, 86)	78(73, 86)	0.698
$PaCO_2$	39(35, 43)	39(36, 43)	39(35, 42)	0.284
T_1				
pH	7.33(7.29, 7.38)	7.33(7.28, 7.37)	7.34(7.29, 7.38)	0.221
Hgb	102(90, 115)	102±17	102±18	0.755
HCT	0.33(0.29, 0.36)	0.33(0.29, 0.36)	0.33(0.29, 0.36)	0.648
Lac	0.7(0.5, 0.8)	0.6(0.5, 0.9)	0.7(0.5, 0.8)	0.847
PaO_2	228 (199, 264)	228±51	239±63	0.087
$PaCO_2$	45(41, 51)	45(41, 52)	44(40, 49)	0.086
T_2				
pH	7.32(7.28, 7.36)	7.32(7.27, 7.36)	7.32(7.28, 7.35)	0.841
Hgb	100±18	99(90, 112)	99(84, 115)	0.758
HCT	0.32(0.28, 0.36)	0.32(0.29, 0.35)	0.32(0.27, 0.36)	0.518
Lac	0.7(0.6, 1.0)	0.7(0.6, 0.9)	0.7(0.5, 1.0)	0.843
PaO_2	221(189, 256)	223±51	220±54	0.588
$PaCO_2$	45(42, 51)	46(42, 51)	45(41, 50)	0.353
T_3				
pH	7.32(7.29, 7.35)	7.32(7.29, 7.35)	7.32(7.29, 7.36)	0.391
Hgb	99(87, 111)	99±16	100±18	0.872
HCT	0.31(0.28, 0.35)	0.32±0.05	0.31±0.06	0.708
Lac	0.8(0.6, 1.0)	0.7(0.6, 1.0)	0.8(0.6, 1.0)	0.151

PaO_2	215(181, 256)	217±51	219±57	0.682
$PaCO_2$	45(42, 50)	45(42, 50)	45(41, 49)	0.904
Postoperative NRS				
Postoperative day 1	3(2, 4)	3(2, 4)	3(3, 4)	0.293
Postoperative day 2	3(2, 4)	3(2, 3)	3(2, 3)	0.093
Postoperative day 3	2(1, 2)	2(1, 2)	2(1, 3)	0.379

Data are presented as mean±standard deviation, median (25th, 75th percentiles), median [min, max], or frequency (column %). Hgb, hemoglobin; HCT, Hematocrit; Lac, lactic acid; PaO_2, arterial partial pressure of oxygen; $PaCO_2$, arterial partial pressure of carbon dioxide; NRS, Numeric Rating Scale.T_0, at room air; T_1, at1 h after surgery; T_2, at 2 h after surgery; T_3, at the end of surgery. N, preoperative lacunar infarcts-negative group; P, preoperative lacunar infarcts-positive group.

Supplementary Table 2. Dynamic changes of intraoperative regional cerebral oxygen saturation between N and P groups.

$rScO_2$	N group (n = 208)	P group (n =161)	*P* Value
T_0	62.2(60.5, 64.3)	60.3(57.8, 62.7)	<0.001
T_1	63.2±3.6	60.6±4.2	<0.001
T_2	64.3±3.3	62.5±4.0	<0.001
T_3	64.3±3.3	62.4±4.0	<0.001
T_4	65.2(62.4, 67.4)	62.9(59.8, 65.6)	<0.001
T_5	65.3±4.1	62.7±4.6	<0.001
T_6	64.6±4.3	62.9±5.0	<0.001

Data are presented as mean±standard deviation, median (25th, 75th percentiles), median [min, max], or frequency (column %). $rScO_2$, regional cerebral oxygen saturation.T_0, at room air; T_1, at the start of induction; T_2, at the end of induction; T_3, the start of surgery; T_4, at1 h after surgery; T_5, at 2 h after surgery; T_6, at the end of surgery; N, preoperative lacunar infarcts-negative group; P, preoperative lacunar infarcts-positive group.

Supplementary Table 3. Backward logistics regression analysis of Model 1

**Model 1** Variables	Univariable OR (95% CI)	*P* value	Multivariable OR (95% CI)	*P* value
Preoperative lacunar infarcts	1.66 (1.07-2.58)	0.023	1.54(0.97-2.43)	0.066
Age				
<75	Ref	-	-	-
≥75	2.06 (1.29-3.28)	0.002	2.00(1.23-3.24)	**0.005**
Sex				
Female	Ref	-	-	-
Male	0.63(0.38-1.03)	0.064	0.58(0.35-0.97)	**0.038**
Preoperative MMSE	0.89 (0.80-0.98)	0.014	0.89(0.80-0.98)	**0.019**
Cerebral desaturation	1.02(0.89-1.18)	0.759	1.86(0.96-3.60)	0.067

OR, odds ratio; CI, confidence interval; MMSE, Mini-Mental State Examination; ASA, American Society of Anesthesiologists Physical Status.

Cerebral desaturation was defined as a reduction in oximetry values by 10% or more from baseline, lasting for 1-minute or longer.

Adjust for age, sex, preoperative MMSE, and cerebral desaturation.

Supplementary Table 4. Logistics analysis of Model 2.

**Model 2**<br>Variables	Univariable<br>OR (95% CI)	*P* value	Multivariable<br>OR (95% CI)	*P* value
Preoperative lacunar infarcts	1.66 (1.07-2.58)	0.023	1.51(0.95-2.40)	0.083
Age				
<75	Ref	-	-	-
≥75	2.06 (1.29-3.28)	0.002	1.98(1.22-3.23)	**0.006**
Sex				
Female	Ref	-	-	-
Male	0.63(0.38-1.03)	0.064	0.58(0.35-0.97)	**0.038**
Preoperative MMSE	0.89 (0.80-0.98)	0.014	0.89(0.80-0.98)	**0.020**
Cerebral desaturation	1.06(0.60-1.87)	0.082	1.90(0.97-3.71)	0.061
Anemia	1.01(0.65-1.57)	0.977	0.88(0.55-1.41)	0.600
ASA physical status	1.46 (0.90-2.37)	0.127	1.37(0.82-2.29)	0.223
Duration of surgery	1.02(0.89-1.18)	0.759	1.03(0.89-1.20	0.690

OR, odds ratio; CI, confidence interval; MMSE, Mini-Mental State Examination; ASA, American Society of Anesthesiologists Physical Status.

Cerebral desaturation was defined as a reduction in oximetry values by 10% or more from baseline, lasting for 1-minute or longer.

Adjust for age, sex, preoperative MMSE and cerebral desaturation, anemia, ASA physical status, and duration of surgery.

Supplementary Table 5. Logistics analysis of Model 3.

**Model 3**	Univariable		Multivariable	
Variables	OR (95% CI)	*P* value	OR (95% CI)	*P* value
Preoperative lacunar infarcts	1.66 (1.07-2.58)	0.023	1.49(0.94-2.37)	0.090
Age				
<75	Ref	-	-	-
≥75	2.06 (1.29-3.28)	0.002	1.97(1.21-3.20)	**0.006**
Sex				
Female	Ref	-	-	-
Male	0.63(0.38-1.03)	0.064	0.58(0.34-0.97)	**0.037**
Preoperative MMSE	0.89 (0.80-0.98)	0.014	0.89(0.80-0.98)	**0.021**
Cerebral desaturation	1.06(0.60-1.87)	0.082	1.89(0.97-3.70)	0.062
ASA physical status	1.46 (0.90-2.37)	0.127	1.38(0.83-2.30)	0.217
Duration of surgery	1.02(0.89-1.18)	0.759	1.03(0.89-1.19)	0.714

OR, odds ratio; CI, confidence interval; MMSE, Mini-Mental State Examination; ASA, American Society of Anesthesiologists Physical Status.

Cerebral desaturation was defined as a reduction in oximetry values by 10% or more from baseline, lasting for 1-minute or longer.

Adjust for age, sex, preoperative MMSE, cerebral desaturation, ASA physical status, and duration of surgery.

Supplementary Table 6. Logistics analysis of Model 4.

**Model 4**<br>Variables	Univariable<br>OR (95% CI)	*P* value	Multivariable<br>OR (95% CI)	*P* value
Preoperative lacunar infarcts	1.66 (1.07-2.58)	0.023	1.55(0.98-2.44)	0.063
Age				
<75	Ref	-	-	-
≥75	2.58 (0.51-13.05)	0.253	2.00(1.23-3.25)	**0.005**
Sex				
Female	Ref	-	-	-
Male	0.86(0.42-1.73)	0.663	0.59(0.35-0.98)	**0.042**
Preoperative MMSE	0.89 (0.80-0.98)	0.014	0.88(0.80-0.98)	**0.019**
Cerebral desaturation	1.06(0.60-1.87)	0.082	1.84(0.94-3.57)	0.074
Duration of surgery	1.02(0.89-1.18)	0.759	1.02(0.88-1.19)	0.782

OR, odds ratio; CI, confidence interval; MMSE, Mini-Mental State Examination; ASA, American Society of Anesthesiologists Physical Status.

Cerebral desaturation was defined as a reduction in oximetry values by 10% or more from baseline, lasting for 1-minute or longer.

Adjust for age, sex, preoperative MMSE, cerebral desaturation, and duration of surgery.

Supplementary Table 7. Adverse clinical outcomes during hospitalization

	N group (n = 208)	P group (n = 161)	*P* value
Acute heart failure	0 (0)	0 (0)	-
Acute myocardial infarction	0 (0)	0 (0)	-
[a] Severe postoperative infections	9 (5.6%)	11 (5.3%)	0.734
Acute stroke	0 (0)	0 (0)	-
[b] Severe postoperative pain	19 (11.8%)	27 (13.0%)	0.899
Death	0 (0)	0 (0)	-

Data are presented as mean±standard deviation, median (25th, 75th percentiles), median [min, max], or frequency (column %). N, preoperative lacunar infarcts-negative group; P, preoperative lacunar infarcts-positive group.

[a] Severe postoperative infections was diagnosed by medical records.

[b] Severe postoperative pain was defined as NRS ≥5.
